# The Mediating Role of Perceived Stress in the Relationship of Self-Efficacy and Work Engagement in Nurses

**DOI:** 10.3390/jcm8010010

**Published:** 2018-12-21

**Authors:** María del Carmen Pérez-Fuentes, María del Mar Molero Jurado, Ana Belén Barragán Martín, María del Mar Simón Márquez, África Martos Martínez, José Jesús Gázquez Linares

**Affiliations:** 1 Department of Psychology, Faculty of Psychology, University of Almería, 04120 Almería, Spain; mmj130@ual.es (M.d.M.M.J.); abm410@ual.es (A.B.B.M.); msm112@ual.es (M.d.M.S.M.); amm521@ual.es (Á.M.M.); jlinares@ual.es (J.J.G.L.); 2 Department of Psychology, Faculty of Psychology, Universidad Autónoma de Chile, 4780000 Santiago, Chile

**Keywords:** stress perceived, self-efficacy, engagement, work, nursing

## Abstract

Positive occupational health psychology (POHP) examines the mechanisms that promote the health and wellbeing of workers, in addition to the risk factors arising from work activity. The aim of this study was to analyse the mediating role of perceived stress in the effect that self-efficacy has on engagement in nurses. The sample was comprised of 1777 currently working nurses. We administered the Utrecht Work Engagement Scale (UWES), the Perceived Stress Questionnaire and the General Self-Efficacy Scale. Following bivariate correlational analysis, multiple linear regression analysis and simple and multiple mediation analysis, the results showed self-efficacy to be a powerful personal resource that positively predicts employee engagement, although the effect diminishes when there are mediating variables of stress. We found differences in the way the different aspects of stress mediated the relationship between self-efficacy and the engagement dimensions. “Energy–joy” was the strongest mediating variable for all of the engagement dimensions and this, together with “harassment–social acceptance” dampened the effect of self-efficacy on vigour and dedication, whereas “Overload” was only a mediator for dedication. As nurses work in a stressful environment, risk factors arise from work activity, so hospital management should design interventions to enhance their workers’ personal resources and improve personal and organizational wellbeing.

## 1. Introduction

Toward the end of the 20th century, occupational health psychology (OHP) appeared as a specialist area of psychology with the aim of “improving the quality of work life and protecting and promoting the safety, health and wellbeing of workers” (National Institute of Occupational Safety and Health; NIOSH) [1]. Historically, psychology has been concerned with negative aspects of health; as Salanova and Schaufeli [2] put it, this discipline has been interested in the study of the 4Ds (Diseases, Disorders, Damage and Disabilities) (e.g., burnout, mobbing, absenteeism in the workplace, musculoskeletal problems) [3]. The appearance of positive psychology led to greater interest in the positive aspects of human functioning [4,5]. This is the context of positive occupational health psychology (POHP), which arose from the concept of integrated health and positive organizational psychology, concerned with “the scientific study of the optimal functioning of the health of individuals and groups in organizations, as well as the effective management of psychosocial well-being at work and the development of healthy organizations.” [1] (p. 23). 

From this perspective, POHP has paid particular attention to the study of engagement or organizational commitment, defined as “a positive, fulfilling, work-related state of mind that is characterized by vigour, dedication and absorption” [6] (p. 74). Vigour is characterized by high levels of energy, effort at work and persistence in the face of difficulties. Dedication refers to strong involvement in the job and having a sense of significance, pride and challenge in the work. Finally, absorption means fully concentrating and being immersed in the work, such that time passes quickly [7].

Engagement as a construct has been widely studied as it has been implicated in various positive results for both workers and organizations. It has been positively related with health [8], happiness [9] and satisfaction [10]. It has also been linked to behaviour, which is beneficial to the organization including personal initiative [11], active learning [12], proactive behaviour such as job crafting [13], customer satisfaction [14], quality of service [15], individual performance [16] and organizational performance [17].

Since its inception, the job demands–resources model (JD–R) [18] has been the reference framework for research into work related wellbeing and stress [19]. This model views job resources as the best indicators of engagement of both individual and organizational performance via a motivational process [20]. The model also highlights the role of workers’ personal resources, defined as positive self-evaluation or belief of control the workers have over their environment, because it is positively related to engagement and performance and also reduces the negative impact of job demands [19,21]. 

In terms of personal resources, the literature has underlined the relationship of workers’ “self-efficacy” with indicators of wellbeing and occupational health [22,23]. In social cognitive theory (SCT) developed by Albert Bandura [24], it is defined as belief in their own abilities to organize and carry out courses of action needed to produce specific future successes. SCT supposes that beliefs of self-efficacy affect forms of behaviour, thinking and feeling. For example, individuals tend to choose tasks that they feel capable of doing, avoiding tasks that are beyond their abilities; in addition, people who feel that they are not very effective in the face of the demands of their surroundings exaggerate their deficits, producing negative thoughts that leads to stress and makes it more difficult for them to use the resources available to them [25,26,27,28]. Much empirical research has looked at the role of self-efficacy in the context of work and has shown that positive belief of self-efficacy predicts positive states such as engagement through gain spirals, especially when the job is demanding [19,29]. Self-efficacy also performs a buffering role in the face of various job demands [30,31,32].

In the job demands–resources model, “stress” is thought of as a demand in the context of work, one that can trigger a process of deterioration of worker health that may be reflected in various mood disorders (e.g., depression) and physical problems (e.g., musculoskeletal or cardiovascular issues) [33]. In fact, stress is one of the main objects of study for occupational health psychology as it is one possible precursor to burnout [20].

Our aim in this study was to evaluate the mediating role of stress in the effect self-efficacy has on engagement in a sample of nursing professionals. Nursing has been the subject of numerous studies due to its reputation for being a particularly stressful profession [34,35] but one of the strengths of our current work is the interest in wellbeing from the perspective of positive occupational health psychology (POHP).

## 2. Materials and Methods

### 2.1. Participants

The sample in our study was made up of a total of 1777 active nursing professionals. The initial sample was made up of 1883 nurses from Andalucía (Spain) who were randomly selected from various centres. We identified 106 cases that were removed from the sample for not completing the entire questionnaire (19 subjects) or because we found that they had completed it randomly (87 subjects). As the main variable in the study was engagement, the selection of participants included noting their current working situation (permanent or temporary contracts). At the time of the study, 71.6% (*n* = 1273) were working under temporary contracts and 28.4% (*n* = 504) were working under permanent contracts. The mean age of the participants was 32.02 (*SD* = 6.69), ranging from 22 to 60 years old. Over four fifths (85.4%, *n* = 1517) were women and the remaining 14.6% were men, with mean ages of 32.01 (*SD* = 6.63) and 32.10 (*SD* = 7.01), respectively. Just over half (51.5%, *n* = 916) of the participants were single, 46.1% (*n* = 819) were married or in a stable relationship, 2.3% (*n* = 40) were divorced or separated and 0.1% (*n* = 2) were widowed. 

### 2.2. Instruments

The Utrecht Work Engagement Scale (UWES) [7] is a self-reported scale for evaluating engagement at work. It contains 17 items with 7-point Likert type responses. It provides information about three aspects of engagement: Vigour, Dedication and Absorption. The scale gives a total *engagement* score and a score for each of the three individual dimensions. This instrument has achieved appropriate levels of reliability and validity [6]. In our sample of nurses, the indexes of internal reliability in each of the dimensions were excellent. The values were 0.84 for Vigour, 0.89 for Dedication and 0.81 for Absorption.

The Perceived Stress Questionnaire from Levenstein et al. [36] was designed specifically to measure stress in psychosomatic clinical research. The original version was made up of 30 elements in six scales: harassment–social acceptance, overload, irritability–tension–fatigue, energy–joy, fear–anxiety and self-realization–satisfaction. In this case, we used the Spanish adaptation of 11 items [33], which demonstrated a general reliability of 0.80 in a research sample of health workers and students. In our case, in the sample of nurses, the instrument gave a general reliability of 0.79. Cronbach’s alpha index for the scales varied between 0.62 and 0.80. 

The General Self-Efficacy Scale [37] is made up of 10 items with 4-point Likert type responses. It evaluates a person’s perception of their personal competence to effectively manage different stressful situations. Authors such as [38] have examined the reliability of the scale and obtained a Cronbach alpha of 0.87. In the current study, in the calculation of the scale’s internal consistency, we obtained an alpha of 0.92.

### 2.3. Procedure

Once the evaluation instruments were selected and before data collection, the participants in the sample were assured that the study would comply with appropriate standards of data retention, confidentiality and ethics in how the data would be treated. The study was approved by the Bioethics Committee at the University of Almería. The questionnaires were applied through a web platform ad hoc, which allowed each subject to complete their part online. In order to check for random or incongruent responses, we included a series of control questions (e.g., I put my shoes on my head every day, with the answer options, It never happens to me, Sometimes it happens to me, It almost always happens to me and it always happens to me), which would detect those cases and highlight anyone in the sample who responded randomly.

### 2.4. Data Analysis 

This study had a quantitative descriptive design. This paper also included valuable recommendations for the revision of Strengthening the Reporting of Observational Studies in Epidemiology (STROBE) [39]. First, the relationships between the variables were examined by the analysis of bivariate correlations. To understand how the predictor variables (self-efficacy; perceived stress: harassment–social-acceptance, overload, irritability–tension–fatigue, energy–joy, fear–anxiety, self-realization–satisfaction) related to the criterion variable (Engagement: Vigour, Dedication and Absorption), we carried out stepwise multiple linear regression.

To check the mediating effect of the variables in each of the regression models, we performed simple and multiple mediation analysis with three mediating variables (for each case, the independent variable was the variable with the greatest explanatory value in the regression model according to standardized coefficients, with the other variables included in the equation considered as possible mediators). The regression models were produced using the SPSS macro for simple and multiple mediation effects by Preacher and Hayes [40,41]. In addition, we applied the bootstrapping technique with coefficients estimated from 5000 bootstrap samples.

## 3. Results

### 3.1. Self-Efficacy, Perceived Stress and Engagement 

As Table 1 shows, self-efficacy was positively correlated with the three engagement dimensions (Vigour: *r* = 0.51, *p* < 0.001; Dedication: *r* = 0.45, *p* < 0.001; Absorption: *r* = 0.38, *p* < 0.001) and was negatively correlated with most of the components of perceived stress (H–SA: *r* = −0.19, *p* < 0.001; I–T–F: *r* = −0.22, *p* < 0.001; E–J: *r* = 0.39, *p* < 0.001; F–A: *r* = −0.29, *p* < 0.001; SR–S: *r* = −0.11, *p* < 0.001). 

In the relationships between the *engagement* dimensions and the components of perceived stress, Vigour was positively correlated with energy–joy (*r* = 0.43, *p* < 0.001) and negatively correlated with the other stress factors (H–SA: *r* = −0.25; *p* < 0.001; SOB: *r* = −0.09, *p* < 0.001; I–T–F: *r* = −0.28, *p* < 0.001; F–A: *r* = −0.26, *p* < 0.001; SR–S: *r* = −0.07, *p* < 0.01). Dedication was positively correlated with energy–joy (*r* = 0.43, *p* < 0.001) while being negatively correlated with: harassment–social acceptance (*r* = −0.29; *p* < 0.001), overload (*r* = −0.08, *p* < 0.001), irritability–tension–fatigue (*r* = −0.28, *p* < 0.001) and fear–anxiety (*r* = −0.25, *p* < 0.001). Finally, Absorption was also positively correlated with energy–joy (*r* = 0.30, *p* < 0.001) and negatively correlated with harassment–social acceptance (*r* = −0.16; *p* < 0.001), irritability–tension–fatigue (*r* = −0.18, *p* < 0.001) and fear–anxiety (*r* = −0.16, *p* < 0.001).

### 3.2. Self-Efficacy and Components of Perceived Stress as Predictors of Engagement in Nurses

Using the correlational analysis data, we performed multiple linear regression analysis with the aim of identifying the predictor variables in each case. Table 2 shows that for the *engagement* dimension of Vigour, the regression analysis gave four models, with the fourth having the greatest explanatory power, with 33.6% (*R*^2^ = 0.33) of the variance explained by the factors included in the model. To confirm the validity of the model, we analysed the independence of the residuals. The Durbin-Watson D statistic gave a value of *D* = 1.97, which confirmed the absence of positive and negative autocorrelation. In addition, the value of *t* was associated with a probability of error of less than 0.05 in all of the variables included in the model. The standardized coefficients showed that the variable with the greatest explanatory weight was self-efficacy. Finally, the values of the tolerance indicators and VIF indicated the absence of collinearity between the variables in the model.

With Dedication, the regression analysis produced four models, with the final model explaining 30.6% (*R*^2^ = 0.30) of the variance. In this case, the Durbin-Watson *D* statistic confirmed the validity of the model (*D* = 1.93). The value of *t* suggested a probability of error of less than 0.05 for all of the variables in the model. The values of the standardized coefficients indicated that self-efficacy was the strongest predictor of Dedication in this sample. The values of the tolerance indicators and VIF indicated the absence of collinearity between the variables. 

Finally, for Absorption, the regression analysis produced two models. with the second explaining 17.9% of the variance (*R*^2^ = 0.17) and a *D* statistic of *D* = 1.95, which confirmed the validity of the model. The value of the *t* statistic suggested an association between the variables with a probability of error of less than 0.05 for all of the variables in the model. Again, self-efficacy was the strongest predictor of this engagement dimension. The values of the tolerance indicators and VIF indicated the absence of collinearity between the variables in the model.

### 3.3. Mediation Models for the Estimation of Predictors and Routes of Mediation Effects for Engagement Dimensions

Following the regression analysis, self-efficacy was identified as the independent or predictor variable and the other variables were included in the model as mediating variables. Three mediation models were generated, each with self-efficacy as the independent variable. In the first, with Vigour as the dependent variable, a multiple mediation model was examined with three mediating variables (M_1_: E–J, M_2_: H–SA and M_3_: SR–S). The second, predicting mediating effects on Dedication, included three mediating variables (M_1_: E–J, M_2_: H–SA and M_3_: SOB). The third, with Absorption as the dependent variable, was a simple mediation analysis with a single mediating variable (M_1_: E–J).

**Figure 1:**
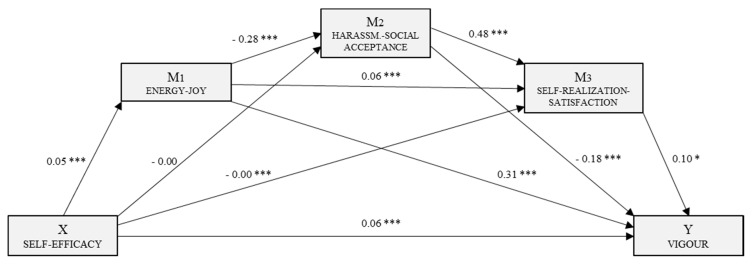
Multiple mediation model of perceived stress in the relationship between self-efficacy and the engagement dimension, Vigour. * *p* < 0.05; *** *p* < 0.001.

Figure 1 shows the multiple mediation model for Vigour including the direct, indirect and total effects. There is a statistically significant effect (*B* = 0.05, *p* < 0.001) of self-efficacy (X) on energy–joy (M_1_). The second regression analysis, with mediator 2 as the outcome variable, included the variables self-efficacy (X) and energy–joy (M_1_). Energy–joy had a significant effect (*B* = −0.28, *p* < 0.001) on harassment–social acceptance (M_2_), which was not the case with self-efficacy (*B* = –0.002, *p* = 0.21). With the third regression analysis, taking self-realization–satisfaction (M_3_) as the outcome variable, we could estimate the effect of the independent variable and the effects of the other two mediators. In each case, we saw significant effects: self-efficacy (*B* = −0.005, *p* < 0.01), energy–joy (*B* = 0.06, *p* < 0.001) and self-realization–satisfaction (*B* = 0.48, *p* < 0.001). In addition, self-efficacy (*B* = 0.06, *p* < 0.001), energy–joy (*B* = 0.31, *p* < 0.001), harassment–social acceptance (*B* = −0.18, *p* < 0.001) and self-realization–satisfaction (*B* = 0.10, *p* < 0.001) had significant effects on Vigour (Y). The overall effect of self-efficacy on Vigour was significant (*B* = 0.08, *p* < 0.001). Finally, an analysis of the indirect effects via bootstrapping produced data supporting significance for route 1 (ind_1_: X→M_1_→Y; *B* = 0.016, SE = 0.002, 95% CI (0.012, 0.021)) and route 4 (ind_4_: X→M_1_→M_2_→Y; *B* = 0.002, SE = 0.008, 95% CI (0.001, 0.004)).

**Figure 2:**
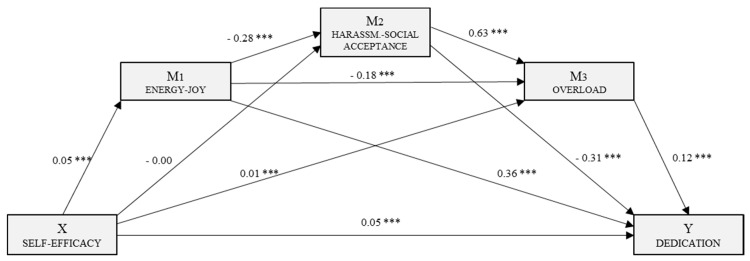
Multiple mediation model of perceived stress in the relationship between self-efficacy and the engagement dimension, Dedication. *** *p* < 0.001.

Figure 2 shows the multiple mediation model for Dedication. Following the third regression analysis, with overload as the outcome variable (M_3_), we estimated the effect of the independent variable and the other mediators. In each case, we saw significant effects: self-efficacy (*B* = 0.01, *p* < 0.001), energy–joy (*B* = −0.18, *p* < 0.001) and harassment–social acceptance (*B* = 0.63, *p* < 0.001). Furthermore, self-efficacy (*B* = 0.05, *p* < 0.001), energy–joy (*B* = 0.36, *p* < 0.001), harassment–social acceptance (*B* = −0.31, *p* < 0.001) and overload (*B* = 0.12, *p* < 0.001) had significant effects on Dedication (Y). The overall effect of self-efficacy on Dedication was significant (*B* = 0.07, *p* < 0.001).

The analysis of the indirect effects via bootstrapping produced data which supported a level of significance for route 1 (ind_1_: X→M_1_→Y; *B* = 0.018, SE = 0.002, 95% CI (0.014, 0.024)), route 3 (ind_3_: X→M_3_→Y; *B* = 0.002, SE = 0.008, 95% CI (0.001, 0.004)) and route 4 (ind_4_: X→M_1_→M_2_→Y; *B* = 0.004, SE = 0.001, 95% CI (0.002, 0.006)).

**Figure 3:**
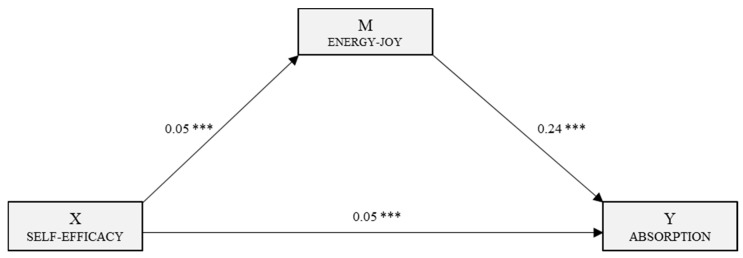
Simple mediation model of perceived stress in the relationship between self-efficacy and the engagement dimension, Absorption. *** *p* < 0.001.

Figure 3 shows the simple mediation model for Absorption. In the first regression analysis, energy–joy (M) was the outcome variable and the effect of self-efficacy was shown to be significant (*B* = 0.05, *p* < 0.001). With the following regression analysis, with Absorption as the outcome variable (Y), we estimated the effects of the independent variable (*B* = 0.05, *p* < 0.001) and the mediator (*B* = 0.24, *p* < 0.001), which were both significant. The overall effect of self-efficacy on Absorption was significant (*B* = 0.06, *p* < 0.001). 

The analysis of the indirect effects via bootstrapping gave a significant effect (*B* = 0.01, SE = 0.002, 95% CI (0.008, 0.017)).

## 4. Discussion

With the appearance of positive occupational health psychology (POHP), the scientific and academic arenas have shown greater interest in the study of the mechanisms that lead to sustainable work-related wellbeing for employees [1,4]. In particular, nurses have been the object of study in much of the empirical research as it is considered to be a very stressful profession and is carried out in a particularly challenging environment in emotional and psychological terms [34,35].

In line with previous research [19,29], the three dimensions of engagement (Vigour, Dedication and Absorption) were positively correlated with self-efficacy. We also found negative correlations between most of the components of stress and self-efficacy and with engagement. In terms of the job demands–resources model, authors such as Bakker et al. [20] indicate that, while self-efficacy may cushion “stress”, it is a job demand that may trigger a process of deterioration in worker health, in addition to being a possible precursor of burnout [19,21]. 

Our results confirmed previous research indicating that self-efficacy is the strongest predictor for all of the dimensions of engagement [19,20]. Self-efficacy beliefs determine our manner of perceiving the work environment in such a way that workers who believe themselves to be effective face challenging workplace demands with effort and perseverance and do not consider failures as indicators of their worth [27]. 

Our data also indicated that the effect of self-efficacy on all of the dimensions of engagement is greater when the relationship is direct and that it diminishes significantly when there are mediating variables of stress. This confirms a partial mediation model.

We found differences in terms of the variables which mediate the relationship between self-efficacy and the different aspects of engagement. “Energy–joy” referring to aspects of wellbeing and health [33], was the strongest mediating variable for all of the engagement dimensions. With a smaller effect, “energy–joy” together with “harassment–social acceptance” were the mediating variables for the Vigour and Dedication dimensions. Finally, “Overload” only appeared as a mediator in the relationship between self-efficacy and Dedication.

The results of our work may have significant practical implications. It is important to highlight the significant effects of self-efficacy on worker and organizational wellbeing, on the promotion of organizational commitment and improving the quality of services, among other issues [22]. Organizations should implement workshops to improve their workers’ personal resources and develop positive interventions to improve job satisfaction, with the aim to enhance employee health [1]. Self-efficacy is a powerful personal resource that predicts nurse participation in the workplace in a positive way but this effect decreases when stress is perceived. Therefore, if we want to improve self-efficacy and the participation of nurses, we must work to reduce their stress levels.

This work is not without limitations, which should be borne in mind when considering the results. First, the data were gathered by self-reporting, which may mean contamination by the common method variance. It would be useful to complement these results with other measures gathered by other methods. Second, the results were not generalizable to the health field as a whole, so it would be interesting to widen the sample to other healthcare professionals. Finally, the transversal design of the study did not allow causal relationships to be established between variables, so it would be advisable to perform longitudinal studies. In the present work, the specialty variable within the nursing profession was not considered, so it could be an aspect to consider in future research.

## 5. Conclusions

The main objective of our study was to evaluate the mediating role of stress in the relationship between self-efficacy and engagement in nurses. This research demonstrated that the strength of the relationship between self-efficacy and engagement diminishes when there are stress-related mediating variables. One of the most important findings in our study is that, while there are differences in terms of the components of perceived stress which mediate the relationship between self-efficacy and aspects of engagement, “energy–joy” was the strongest mediating variable for Vigour, Dedication and Absorption.

This research contributes to the understanding of the importance of self-efficacy in the framework of positive occupational health psychology (POHP) as it deals with a personal resource which acts as a buffer against job demands and significantly influences the wellbeing and occupational health of workers.

Future lines of work should continue to explore the topic. It would be interesting to include other personal resources (e.g., self-esteem) and other aspects related to job demands (e.g., role conflict) and others risk factors arising from work activity. The mix of variables should be widened to include aspects related to job resources (e.g., leadership) to complete the structure of the job demands–resources model and thus offer a better understanding of wellbeing at work.

## Figures and Tables

**Table 1:** Self-efficacy, perceived stress and engagement. Bivariate correlations.

Dimensions	Dimensions								
	1	2	3	4	5	6	7	8	9
1. Self-efficacy	–								
2. Harassment–social acceptance	−0.19 ***	–							
3. Overload	−0.02	0.51 ***	–						
4. Irritability–tension–fatigue	−0.22 ***	0.69 ***	0.66 ***	–					
5. Energy–joy	0.39 ***	−0.42 ***	−0.32 ***	−0.52 ***	–				
6. Fear–anxiety	−0.29 ***	0.55 ***	0.47 ***	0.69 ***	−0.45 ***	–			
7. Self-realization–satisfaction	−0.11 ***	0.45 ***	0.42 ***	0.51 ***	−0.14 ***	0.50 ***	–		
8. Vigour	0.51 ***	−0.25 ***	−0.09 ***	−0.28 ***	0.43 ***	−0.26 ***	−0.07 **	–	
9. Dedication	0.45 ***	−0.29 ***	−0.08 ***	−0.28 ***	0.43 ***	−0.25 ***	−0.03	0.84 ***	–
10. Absorption	0.38 ***	−0.16 ***	−0.04	−0.18 ***	0.30 ***	−0.16 ***	−0.01	0.82 ***	0.77 ***

** *p* < 0.01; *** *p* < 0.001.

**Table 2:** Engagement dimensions. Stepwise multiple linear regression model (*N* = 1777).

	Model	*R*	*R* ^2^	Corrected *R*^2^	Change Statistics							Durbin Watson
					Typical Error of Estimation	Change in *R*^2^	Change in *F*		Sig. of Change in *F*			
Vigor	1	0.51	0.26	0.26	0.65	0.26	635.29		0.000			1.97
	2	0.57	0.33	0.32	0.62	0.06	175.51		0.000			
	3	0.57	0.33	0.33	0.62	0.00	11.39		0.001			
	4	0.58	0.33	0.33	0.62	0.00	6.24		0.013			
	Model 4		Non-standardized coefficients		Standardized coefficients		*t*	Sig.	Collinearity			
			*B*	Std. Error	Beta				Tol.			VIF
	(Constant)		0.85	0.16			5.22	0.000				
	Self-efficacy		0.06	0.00	0.40		19.12	0.000	0.84			1.19
	Energy–Joy		0.31	0.02	0.24		10.73	0.000	0.71			1.40
	H’ment–Soc. Accpt.		–0.18	0.04	–0.09		–4.13	0.000	0.66			1.51
	S-realization–Satisf.		0.10	0.04	0.05		2.49	0.013	0.78			1.27
Dedication	Model	*R*	*R* ^2^	Corrected *R*^2^	Change statistics						Durbin Watson	
					Typical error of estimation	Change in *R*^2^	Change in *F*		Sig. of change in *F*			
	1	0.45	0.20	0.20	0.70	0.20	469.37		0.000		1.93	
	2	0.53	0.28	0.28	0.66	0.07	196.62		0.000			
	3	0.54	0.30	0.29	0.66	0.01	29.95		0.000			
	4	0.55	0.30	0.30	0.66	0.00	15.24		0.000			
	Model 4		Non-standardized coefficients		Standardized coefficients		*t*	Sig.	Collinearity			
			*B*	Std. Error	*B*				Tol.		VIF	
	(Constant)		1.48	0.16			8.85	0.000				
	Self-efficacy		0.05	0.00	0.32		14.67	0.000	0.82		1.21	
	Energy–Joy		0.36	0.03	0.27		11.52	0.000	0.69		1.44	
	H’ment–Soc.Accpt.		–0.31	0.04	–0.16		–6.65	0.000	0.65		1.51	
	Overload		0.12	0.03	0.09		3.90	0.000	0.70		1.41	
Absorption	Model	*R*	*R* ^2^	Corrected *R*^2^	Change statistics					Durbin Watson		
					Typical error of estimation	Change in *R*^2^	Change in *F*		Sig. of change in *F*			
	1	0.38	0.15	0.15	0.72	0.15	314.64		0.000	1.95		
	2	0.42	0.17	0.17	0.71	0.02	61.37		0.000			
	Model 2		Non-standardized coefficients		Standardized coefficients		*t*	Sig.	Collinearity			
			*B*	Std. Error	*B*				Tol.	VIF		
	(Constant)		1.07	0.12			8.44	0.000				
	Self-efficacy		0.05	0.00	0.31		13.47	0.000	0.84	1.18		
	Energy–Joy		0.24	0.03	0.18		7.83	0.000	0.84	1.18		
